# Inflammation and Autophagy: A Convergent Point between Autism Spectrum Disorder (ASD)-Related Genetic and Environmental Factors: Focus on Aluminum Adjuvants

**DOI:** 10.3390/toxics10090518

**Published:** 2022-08-31

**Authors:** Loïc Angrand, Jean-Daniel Masson, Alberto Rubio-Casillas, Marika Nosten-Bertrand, Guillemette Crépeaux

**Affiliations:** 1 Univ Paris Est Créteil, INSERM, IMRB, F-94010 Créteil, France; loic.angrand@vet-alfort.fr (L.A.); jean-daniel.masson@vet-alfort.fr (J.-D.M.); 2 Ecole Nationale Vétérinaire d’Alfort IMRB, F-94700 Maisons-Alfort, France; 3 INSERM UMR-S 1270, 75005 Paris, France; marika.nosten-bertrand@inserm.fr; 4 Sorbonne Université, Campus Pierre et Marie Curie, 75005 Paris, France; 5 Institut du Fer à Moulin, 75005 Paris, France; 6 Biology Laboratory, Autlán Regional Preparatory School, University of Guadalajara, Autlán 48900, Jalisco, Mexico; alberto110966@gmail.com; 7 Autlán Regional Hospital, Health Secretariat, Autlán 48900, Jalisco, Mexico

**Keywords:** neurodevelopmental disorders, autism spectrum disorder, environment, immune system, autophagy, early exposure, aluminum adjuvants, neurotoxicity

## Abstract

Autism spectrum disorder (ASD), schizophrenia, and bipolar disorder are genetically complex and heterogeneous neurodevelopmental disorders (NDDs) resulting from genetic factors and gene-environment (GxE) interactions for which onset occurs in early brain development. Recent progress highlights the link between ASD and (i) immunogenetics, neurodevelopment, and inflammation, and (ii) impairments of autophagy, a crucial neurodevelopmental process involved in synaptic pruning. Among various environmental factors causing risk for ASD, aluminum (Al)-containing vaccines injected during critical periods have received special attention and triggered relevant scientific questions. The aim of this review is to discuss the current knowledge on the role of early inflammation, immune and autophagy dysfunction in ASD as well as preclinical studies which question Al adjuvant impacts on brain and immune maturation. We highlight the most recent breakthroughs and the lack of epidemiological, pharmacokinetic and pharmacodynamic data constituting a “scientific gap”. We propose additional research, such as genetic studies that could contribute to identify populations at genetic risk, improving diagnosis, and potentially the development of new therapeutic tools.

## 1. Introduction

Autism spectrum disorder (ASD), bipolar disorder (BD), and schizophrenia (SZ) are exceptional neurodevelopmental disorders due to their complex diagnosis and plethora of symptoms, multiple associated comorbidities, and diverse etiology. They exhibit all the obstacles and limitations that complicate genetic mapping, such as genetic heterogeneity, pleiotropy, high frequency of disease-causing alleles, and epigenetic factors [1,2,3,4,5,6,7].

Interestingly, substantial evidence supports the influence of environmental factors in early brain maturation contributing to long-lasting neural and cognitive impairments. Either by direct toxicity or altered genetic regulation, such factors play a key role in the increased prevalence of neurodevelopmental disorders reported in recent decades.

Epidemiological studies have suggested that specific dysregulations and/or activation of the maternal immune system during pregnancy are crucial environmental risk factors for NDD outcomes in the child. Maternal infections can be vertically transmitted to the infant and cause congenital brain infections. Moreover, non-transmissible maternal infections can also indirectly impact fetal brain development and increase the risk of NDDs, through maternal immune activation (MIA) [8,9].

The role of direct early immune activation on the developing organism and NDD development has been extensively described [10,11,12], and the molecular and cellular mechanisms that mediate immunity-related neurodevelopmental alterations are gradually coming to light [13].

To research the effect of successive and cumulative adverse impacts, “multiple hit” models [14,15] have become standard practice, integrating both genetic and environmental factors potentially contributing to the vulnerability of developing these disorders. Preclinical “multiple hit” studies—such as successive perinatal immune activations—have already confirmed the increased risk to progeny and largely contributed to unraveling the molecular and cellular mechanisms that mediate immunity-induced brain alterations during development [16,17]. Another similar model considered the genetic background as the first hit: the individual’s genetic factors interact with the early-life exposome (including persistent organic pollutants), which can be considered by itself a second hit [18]. These two hits combine to form a dormant phenotype that could likely be codified in the epigenome and is vulnerable to a third environmental hit later in life [18].

These observations resemble the well-described phenomenon of microglial priming, the exaggerated inflammatory state and response from glial cells to their microenvironment [19]. Primed glial cells maintain a more active-like morphology (e.g., amoeboid or reactive) in reaction to a baseline stimulus, such as infection, trauma, or aging. However, compared to acutely activated glia, primed cells do not release cytokines and other pro-inflammatory molecules on a long-term basis. Instead, when confronted with a challenge, primed cells generate more cytokines in the brain than unprimed cells [20].

Among the variety of environmental factors suspected to contribute to the pathophysiology of NDDs, repeated early exposures to aluminum (Al)-containing vaccines during critical developmental periods of both nervous and immune systems has received unique attention. In the past five decades, growing concerns have been raised among the scientific community and the general public, through sometimes counter-productive conflicts, on the effects and safety of these compounds within the organism. Among these questions, adjuvant kinetics and potential chronic adverse effects, especially when given early in life, are noteworthy [21,22,23,24]. Of note, the epidemiological studies evaluating any links between vaccination and NDDs did not specifically address Al adjuvant exposure, since most of them focused on Al-free vaccines (measles, mumps and rubella, MMR) [25,26,27]. The increased use of Al adjuvants in vaccines administered before the age of 12 months and the growing evidence of the potential neurotoxicity of Al adjuvants found so far lead us to recall the evolution of Al-containing vaccines. We review pre-clinical and clinical studies questioning their role in neuro-immune interactions during brain development in the context of ASD. Indeed, ASD persistently ranks at the top of NDDs with regard to the relative genetic contribution, including epigenetic and gene-environment (GxE) interactions [28].

## 2. Autism Spectrum Disorder

### 2.1. Definition and Prevalence

ASD is an increasingly common neurodevelopmental disorder, symptomatically and etiologically heterogeneous, defined by core deficits in social communication and the presence of restricted and stereotyped behaviors [29,30]. In children under the age of five, ASD is the leading cause of disability [31]. Boys are three to four times more prone than girls to developing ASD [32,33].

World-wide large-scale investigations estimate that the global prevalence of ASD is 1–2% [34,35]. More specifically, based on the most recent available statistics from the U.S. Centers for Disease Control and Prevention (CDC), tracking for this since 1996, ASD in the United States affects 1 child in 44 (2021 figure for the year 2018) [36], while the prevalence of ASD in Europe varies from 0.44% to 1.97% of children aged 7–9 years old (i.e., from 1 in 51 children to 1 in 227 children), with an average calculated prevalence of 1.22% (i.e., 1 in 89 children) [37]. In Asia, a meta-analysis of published data found that the pooled calculated ASD prevalence is growing, calculated at 0.36% (i.e., 1 in 278 children) [38].

Apart from current prevalence values, monitoring studies report a rapid rise in ASD prevalence over the past several decades [39]. Indeed, current U.S. data are more than three times higher than the first prevalence estimation of 1 in 150 children between 2000 and 2002 [36]. This increase is not fully explained by the evolution of diagnostic criteria, and the age at which children are diagnosed with ASD remains unchanged, at around 50 months [39]; the increase is rather in favor of a role played by environmental risk factors [33,37].

### 2.2. Genetics

Over the past 20 years, despite the extraordinary degree of etiological heterogeneity, the search for ASD genes has been remarkably successful. More than 100 large-effect, rare (often de novo) mutations have been identified in the coding genome. At present, microarray and whole-exome sequencing studies focus on rare variants with convincing statistical support for the association of about a dozen copy number variants (CNVs) and more than 100 genes, a number which is rapidly expanding. More recent studies involving extensive case-control cohorts successfully identified associations with common risk alleles of modest effect, making possible the quantification of cumulative common genetic risks (polygenic risk score) to address polygenic inheritance. Moreover, substantial evidence shows that certain environmental factors could lead to altered epigenetic marks, increasing the risk of neurodevelopmental outcomes associated with ASD and their comorbidities [28,40].

### 2.3. Immune Dysfunction

The link between immunogenetics, inflammation, and ASD is particularly well substantiated. For instance, perinatal maternal infections have long been recognized as a prominent risk factor for the development of ASD in the child [41,42,43], raising the potential contribution of early immunological activation [44]. Immune compounds such as cytokines and chemokines and the cells that produce them in the central nervous system (CNS), particularly microglia, are known to have an important function in normal brain maturation [19]. Furthermore, a causal link is demonstrated between ASD and increased cellular production of Interleukin-6 (IL-6) [45,46] and IL-17 [47,48,49] upon immune activation. Increased levels of inflammatory cytokines in cerebrospinal fluid of ASD patients as well as neuroinflammation in post-mortem brain from ASD individuals were also described [45]. In this context, a subset of 35 adult patients with high-functioning ASD present a chronic natural killer cell inflammatory/activation process, suggestive of cellular hyperactivation [50]. In addition, upregulation of NLRP3 inflammasomes and overproduction of pro-inflammatory cytokines (IL-1β and IL-18) have been described in peripheral blood mononuclear cells (PBMCs) of ASD children compared to controls [51].

Despite existing limitations between human and animal models, preclinical studies significantly contribute to shedding light on molecular and cellular mechanisms that mediate immune-related aspects of normal and pathological brain development [52]. In addition, rodent studies demonstrated that immune dysfunction, including central and peripheral inflammation during perinatal periods (neuroinflammation, increased production of inflammatory cytokines or antibodies, immune cell activation and autoimmunity), impacts the neurodevelopmental trajectory of key circuits in the pathophysiology of ASD [46,53,54,55,56,57,58,59,60]. More recently, both clinical and preclinical studies highlighted the implication of the complement system—a key player in innate immunity—in NDDs, including ASD [12,61]. As a result, a new paradigm has emerged in the field of “immuno-neuropsychiatry”, describing a persistent immunological dysregulation in the pathogenesis of a wide range of neuropsychiatric disorders (for a review, see [62]).

### 2.4. Immune System and Environment: A Convergent Point

Recent major breakthroughs in ASD suggest that the immune system acts as a convergence point between ASD-related genetic and environmental risk stressors [63]. The immune system is our connection with the outside world and, as a result, environmental influences that affect the maternal, fetal, and/or neonatal immune pathways could cause distinct neuroimmune alterations in the developing individual [19]. Immune system activation resulting from exposure to pro-inflammatory external compounds during critical periods could cause permanent effects and increase the risk of NDDs, suggesting that inflammation itself represents an early environmental stressor [64].

This leads to the question of how inflammation-related pathways, including autophagy (see below), might be responsible for the impacts of early environmental factors in ASD, especially in the context of genetic susceptibility.

## 3. Autophagy

Autophagy is a highly regulated and conserved cellular mechanism, necessary for cell survival. By promoting the recycling of organelles and long-lived proteins [65], it preserves cellular homeostasis in conditions of environmental stress. Moreover, it controls the disposal of intracellular pathogens and promotes adaptive immunity [66]. The three main forms (microautophagy, chaperone-mediated autophagy, and macroautophagy) [65] and their dysregulation have been widely described in a broad spectrum of human diseases, along with inflammatory abnormalities (for a review, see [67]). Indeed, autophagy is essential for the genesis and progression of inflammation and immune response [68,69], both regulating one another in a bidirectional way [70,71,72].

### 3.1. Neuronal and Microglial Autophagy

Neurons have developed extremely specialized processes to control autophagy [73]. Neuronal autophagy is essential for early synaptic pruning, the developmental process whereby over 70% of postnatal net spines are eliminated during the normal course of brain maturation to ensure the relevant formation of appropriate neuronal connections [74,75,76]. Local regulation of autophagy is also crucial for proper axon guidance, vesicular and neurotransmitter release, dendritic spine structure, spine pruning, synaptic plasticity, and behavioral outputs of neural networks [74]. When impaired, neuronal autophagy has been linked to distinct brain alterations, particularly neurodegenerative diseases such as Parkinson’s and Alzheimer’s diseases, and neurodevelopmental disorders such as ASD [73,74,77,78].

In addition, up to 15% of all CNS cells are microglia, which are the principal resident immune cells of the brain and spinal cord [79]. Autophagy is a crucial intracellular mechanism enabling the polarization of macrophages and microglia [80] and regulating the level of activation in microglia [70,79]. In fact, microglial autophagy can be either pro- or anti-inflammatory, e.g., by reducing NLRP3 inflammatory components [70,72]. Moreover, the interaction between microglial inflammatory reaction and microglial autophagy is involved in both acute and chronic CNS injuries [70].

Of note, microglial cells are sensitive to peripheral inflammatory stimuli, as observed in mice administered with inflammatory stimuli such as bacterial lipopolysaccharides (LPS). This causes acute long-term regulation of microglial responses in the brain, as well as variable epigenetic remodeling, which lasts at least six months [81]. Furthermore, in a special context of an amyotrophic lateral sclerosis model, modifying macrophages at the periphery has the capacity to change microglial inflammatory reactivity and to modulate the synaptogenesis signaling pathway by microglia [82].

### 3.2. Autophagy Disruption and ASD

Converging clinical studies suggest a role for autophagy in ASD etiology:

1. Enrichment analysis indicated that in genome-wide association studies, risk genes for brain disorders, including ASD, are over-represented in autophagy-related pathways identified in gene ontology biological processes [74]. Another observation is the implication of functionally relevant polymorphisms in autophagy-associated genes in the vulnerability to autoimmune and inflammatory disorders known to be associated with ASD [83,84].

2. Whereas no differences were observed in dendritic spine density of childhood post-mortem ASD versus control brains, the decrease in spine density through adolescence was greater in controls (~45%) than in ASD patients (~16%), demonstrating a developmental defect in net autophagy-related spine pruning in ASD [76]. These endophenotypes resemble the “intense world syndrome” describing the autistic brain as hyper-reactive with a hyper-connectivity of local neural circuits. Such complex connections are characterized by exaggerated neural information processing and storage within the brain microcircuits, caused by a higher number of synaptic connections and increased spine density [76,85].

Similarly, in preclinical studies:

1. Impaired microglial autophagy pathways studied in mice led to defective synaptic pruning, which becomes visible by an abnormally high dendritic spine density [86].

2. Interestingly, rapamycin, an autophagy inducer, rescued social interaction impairments in adolescent mice exposed to in utero valproic acid (an inducer of autism-like behaviors) [87]. Indeed, rapamycin also rescued altered gene expression, highlighting the role of autophagy and the mammalian target of the rapamycin (mTOR) pathway in ASD, suggesting the interest in new therapeutic targets involving autophagy modulation [87].

### 3.3. Autophagy and Blood–Brain Barrier (BBB)

The essential role of autophagy, BBB integrity, and their crosstalk are the topic of several recent studies, especially those related to pathological or toxicological conditions such as ischemia, experimental traumatic brain injury, chronic cerebral hypoperfusion, and metal or metallic nanoparticle toxicity [88,89,90,91,92,93,94].

Recent evidence suggests that (i) induction of the autophagy signaling pathway and decrease in BBB damage (i.e., improvement of BBB integrity) may concomitantly lead to alleviated cognitive impairment in a context of environmental enrichment [93], and (ii) rapamycin produces a neuroprotective effect by modulating neural autophagy and by acting on the mTOR pathway in other cells of the neurovascular unit related to the BBB [95]. Furthermore, in BBB endothelium infections produced by *Streptococcus* B, autophagy could operate as a BBB cell defense strategy in response to invasive and toxin-producing bacteria [96]. It has been shown that BBB function is restored in mice by inhibiting the NLRP3 inflammasome and inducing autophagy at the same time [97].

Autophagy and BBB integrity may also be associated in the context of toxic injury. For instance, metal-containing nanoparticles may typically cross or bypass the BBB, enter the CNS, and induce neurotoxicity, resulting in cognitive dysfunction. Such effects involve glial activation, inflammatory discharge, reactive oxygen species production, and autophagy dysregulation in glial cells [98].

### 3.4. Autophagy and Microbiota

Beyond the key role of autophagy at the level of CNS, another function of this cellular process seems to be relevant in the field of NDDs. Indeed, a clear role has emerged for autophagy in intestinal homeostasis, affecting cell metabolism, as well as proliferative and regenerative capacity [99]. These recent data are particularly interesting considering that (i) the gut microbiota influences brain function through the neuroendocrine, neuroimmune, and autonomic nervous systems and via microbiotic toxin production, and (ii) both gut microbiota and inflammation could have a key role in the pathophysiology of NDDs [100,101,102].

## 4. Al Adjuvants as Potential Environmental Stressors

### 4.1. Vaccinal Policy, Safety, and Al-Based Adjuvants

Vaccination is one of the greatest achievements in medical history, promoting prevention and sometimes the complete eradication of lethal infectious diseases [103,104]. Although traditional vaccines are widely used and tolerated by a vast majority of people, vaccine safety in specific groups of the population has been a matter of concern for the past 50 years, particularly those including ABAs [23,105,106,107,108]. We cite in the following sections different studies indicating potential problems from a historical point of view.

Al was first used for immunization in 1932 to boost the antigen’s immune responses, and it remained the only approved adjuvant used for vaccines for nearly 70 years [109]. In human vaccines, three Al salts are now used as adjuvants: Al oxyhydroxide (Alhydrogel^®^), Al hydroxyphosphate (AdjuPhos^®^), and Al hydroxyphosphate sulfate. These salts are found in approximately 60% of human vaccines [106], and in France, they are included (alone or mixed) in 8 of the 11 mandatory children’s vaccines [110].

**Figure 1:**
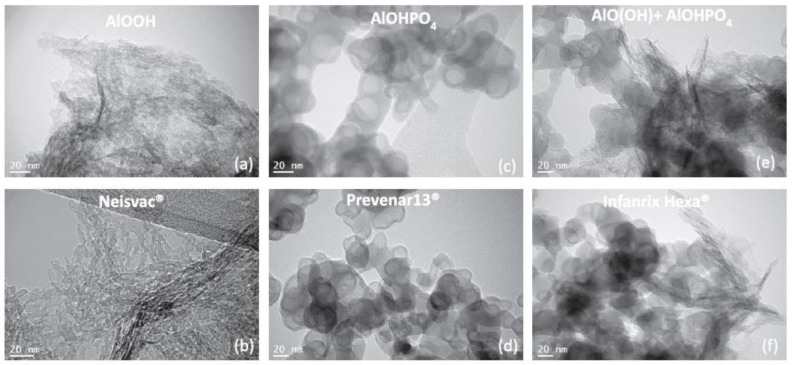
TEM analysis of Al particles in commercial suspensions (**a**,**c**,**e**) and their related vaccines (**b**,**d**,**f**). (**a**,**b**) Al oxyhydroxide, (**c**,**d**) Al hydroxyphosphate, and (**e**,**f**) mix of both Al adjuvants. Reprinted from [112].

Al oxyhydroxide has a positive charge and it is made up of 2.2 nm × 4.5 nm × 10 nm nanoparticles that spontaneously create micron-sized agglomerates with a nano-fibrous aspect when observed with electron microscopy (Figure 1) [111,112]. Alternatively, Al hydroxyphosphate is amorphous and has a slightly negative charge [111] (for a review, see [106]). The different physico-chemical characteristics of these two adjuvants (shape, size, and charge) can strongly influence their biodisposition kinetics—Al oxyhydroxide is less solubilized, more absorbed, and produces a lower toxicity to phagocytic cells than Al hydroxyphosphate [111]—as well as their cellular effects. The rate of aggregation can vary according to these properties and their biochemical environment [112,113]. Consequently, the size of the injected particles modifies their cellular uptake by immune cells and the induced cytotoxicity [111,114] or even affects their ability to translocate in the body.

Reports of negative effects after the use of ABA-containing vaccines started in the 1970s, first describing local reactions (erythema, subcutaneous nodules, contact hypersensitivity, and granulomatous inflammation) [115,116,117]. In the 1990s, the first indications of potential post-immunization development of myalgic encephalomyelitis/chronic fatigue syndrome (ME/CFS), a multifactorial and poorly understood disabling disease, emerged in Canada following the first campaign of immunization against hepatitis B virus (HBV) with Al-adjuvanted vaccines [106,118,119]. Meanwhile, insights came from the Gulf War Illness, pointing out a link between ME/CFS and multiple vaccine administrations within a short period of time, when comparing vaccinated versus non-vaccinated veterans [120,121].

In the late 1990s, reports of macrophagic myofasciitis (MMF), an unusual side effect to ABA-containing vaccines, described a muscular histopathological lesion associated with arthromyalgia, chronic fatigue, and cognitive impairments [21,122,123]. Interestingly, a persistency of Al oxyhydroxide particles within immune cells occurred in both adults and children [21], questioning the kinetics of these compounds in the body [124,125,126,127,128,129].

In the following decade, the term “autoimmune/inflammatory syndrome induced by adjuvants (ASIA)” was proposed to describe a condition where the exposure to an adjuvant (for instance, ABAs) leads to an aberrant autoimmune response [22,130].

Those observations occurred in a context where (1) toxicological studies on Al were extensively documented [131,132,133], and (2) environmental Al was suspected to be a cofactor in a variety of chronic disorders [134]. In addition, Al neurotoxicity was reported in 1997 in preterm infants, showing that prolonged intravenous feeding with soluble Al-containing solutions leads to neurological development impairments [135]. These studies addressed only soluble Al compounds (mainly present in food or in drinking water), yet they contributed to a general concern by the World Health Organization (WHO) and the French National Academy of Pharmacy concerning ABAs’ potential toxicity and the need for further research on Al adjuvants’ pharmacokinetics and safety [136,137,138].

### 4.2. ABAs in Vaccines

Although the toxicity and kinetics of Al and soluble Al compounds are well described [132,133,139], not much information is available regarding ABAs. Indeed, pharmacokinetic data from in vivo as well as mathematical models, useful to predict Al concentrations in plasma and tissue after vaccination in children and adults, are missing, and this constitutes a “scientific gap” [140,141]. Moreover, any attempt to extrapolate ABAs’ fate from soluble Al data is misleading since the frequency of exposure, the route of administration, and the form of Al are different [132]. In particular, (i) when injected intramuscularly, Al oxyhydroxide is assimilated at nearly 100% efficacy over time, whereas dietary absorption is less than 1% [107,142], and (ii) the adjuvant essentially remains immune cell-bound after injection. Indeed, it was shown that instead of fast elimination through urine (as previously claimed) [143], Al oxyhydroxide is subjected to remarkable retention within the body [144]. Al oxyhydroxide nanoparticles spontaneously form aggregates which after injection are promptly engulfed, remaining within phagocytic cells for long periods in both humans and animals [21,145]. Furthermore, Al particles can migrate, through monocyte/macrophage cells, from the injection site to distant organs [106,123,145,146,147,148].

In the 1960s, the Food and Drug Administration established the approved amount of 0.85 mg Al per dose of vaccine based on adjuvant efficiency [149], without providing results from potential safety protocols. In addition, the simultaneous administration of several Al-containing vaccines was not considered, nor were post-injection kinetics or toxicity levels [150]. Finally, as mentioned above, the argument stating that doses of ABAs are low compared to Al daily dietary exposure is worthless, considering the early, acute, and repeated exposure to vaccines [150,151] and the different pharmacokinetic properties of soluble versus particulate Al.

Children across the world receive significant doses of vaccines throughout their life. *Haemophilus influenzae* type b, hepatitis B, *pneumococcal*, DTaP (*diphtheria*, *tetanus*, and *pertussis*), and hepatitis A vaccines are all injected in early childhood in the United States and Europe, and ABAs are present in each of those [110,151,152,153]. In the United States, the first injection with the hepatitis B vaccine may occur on the first day of life [153], reaching at least thirteen Al-containing vaccine injections by the age of 18 months (i.e., a total dose of almost 3 to 4 mg Al/infant) [154]. Our recent study focusing on the 2018 French vaccination schedule showed that (i) half of the exposure occurs before 1 year of age; (ii) an adult following vaccination requirements and recommendations receives between 2.5 and 7.7 mg of Al^3+^ during his/her lifetime; (iii) exposure varies according to age, weight, sex, and selection of one vaccine among several for the same valence [110]. From the end of the 1990s, an increase of 25% to Al exposure through vaccination from birth to 18 months of age has been reported [154].

However, the real amount of Al used in different vaccines is not controlled by regulatory agencies. Additionally, a study evaluating the Al content of thirteen vaccines showed significant variability between batches which did not correspond to the stated amount by the manufacturer (up to 0.602 mg/vaccine for Havrix, a hepatitis A vaccine from GSK, assumed to contain 0.25 mg of Al) [155].

Worth noting is the long-term persistence of Al oxyhydroxide aggregates in immune cells. In addition to evoking a long-lasting immune stimulation, it allows slow adjuvant translocation to remote lymphoid organs and to the brain [123,145,146,147]. Al adjuvants can cross the BBB and may induce immunoinflammatory responses in neural tissues: this translocation has been observed in animals exposed to ABAs, Al-containing vaccines, or ABA-trackers through intramuscular, subcutaneous, or intraperitoneal injections [143,145,146,147,156,157,158,159]. These findings prompted Khan et al. to conclude that repetitive doses of Al oxyhydroxide are “insidiously unsafe”, particularly when given to newborns with an immature BBB. In addition, ABA removal from the CNS is considered to be nearly impossible [146].

Finally, experimental studies focused on the biopersistence and neurotoxic effects of these compounds addressed in different animal models (mainly rodents, rabbits and sheep) showed that ABAs (mainly Al oxyhydroxide) or Al-containing vaccines (i) are capable of inducing behavioral alterations [157,160,161,162,163,164,165,166,167,168,169], (ii) remain in the organism [143,145,146,147,158,170,171,172,173,174,175,176,177,178], and (iii) can leave the injection area to reach remote organs such as the nervous system [146,157,163,164,173,179,180]. Of these thirty-one studies, only six evaluated perinatal period exposure: two studies on gestational exposure on rats [181,182], three studies on newborn mice [163,167,169], and one on newborn rats [183] (for a review, see [148]).

### 4.3. Biological Effects of ABAs on the Immune System

No agreement has been reached regarding the mechanism of action and function of ABAs in distinct features of immune modulation, constituting a long-standing topic in the area of vaccination and immunology [184,185]. In vitro studies have shown that these adjuvants are able to slowly release the antigen in which they are adsorbed and therefore promote the immune response in the presence of even poorly immunogenic antigens [186]. At early stages, ABAs are also capable of stimulating the differentiation of monocytes into dendritic cells [187] and enhancing antigen uptake by antigen-presenting cells, including dendritic cells and macrophages, resulting in antigen phagocytosis [188]. They also have the ability to activate the inflammasome pathway [189,190], leading to the release of mature IL-1β and IL-18 by dendritic cells and to the differentiation of T helper 2 (Th2) cells, thus stimulating B cell activation and antibody release, notably IgG [185,191].

These adjuvants, together with other Al components such as water-soluble Al, are able to induce increased levels of IL-6 in blood and distant organs (i.e., kidney and brain) in both juvenile and adult rodents [183,189,192,193,194,195,196,197,198]. This effect occurs possibly in response to the oxidative stress induced by Al [192,199].

Al oxyhydroxide and Al hydroxyphosphate are Th2 adjuvants that might operate synergically with elements to cause a Th1 to Th2 transition in adaptive T cell reactions, such as psychological stress, excessive sympathetic stimulation, large amounts of corticosteroids, high female hormone production, immune suppression, chronic infection, or overwhelming microbial burden [200,201,202].

Moreover, cerebral absorption of ABA-loaded cells has been demonstrated to be greatly favored by macrophage chemotactic protein-1 (MCP1)/CCL2 expression, which is boosted by Al oxyhydroxide [203] and presumably polarizes vaccination response favoring Th2 immune responses [204]. Interestingly, inflammation is elicited by Al oxyhydroxide particles by triggering the NALP3 inflammasome [205]. The main characteristic of this activation process is the release of IL-1β, which was detected in both brain immune cells and neurons loaded with Al oxyhydroxide particles in mouse experiments [146].

**Figure 2:**
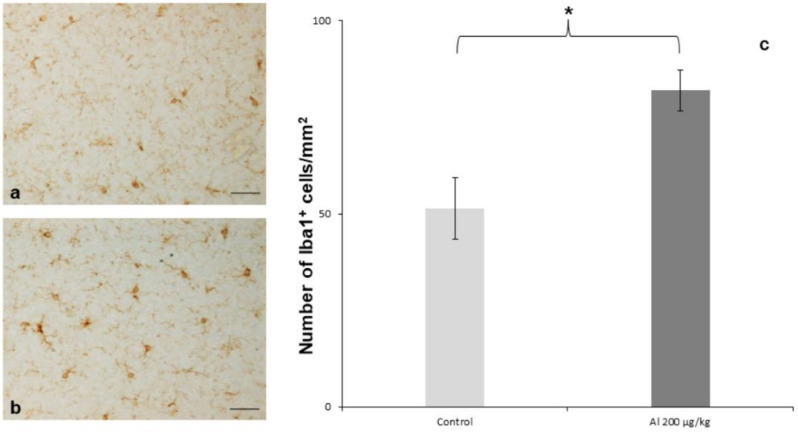
Iba1+ microglial cell density in the ventral forebrain. Iba-1 immunostaining showed a slight increase in the microglial cell density in the group of mice injected with Alhydrogel^®^ 200 µg Al/kg. (**a**) Control mice injected with PBS; (**b**) mice injected with Alhydrogel^®^ 200 µg Al/kg; (**c**) quantification of the microglial cell density. Three mice/group; results expressed as mean ± S.E.M, ANOVA test with post-hoc Bonferroni test, * *p* < 0.05; scale bars: 50 µm. Reprinted and adapted from [164].

Rodent studies have also shown that exposure to ABAs or Al-containing vaccines can lead to microglial activation or increase pro-inflammatory cytokine levels (for a review, see [206]). Microglial activation—suggesting an ongoing inflammatory process—was observed 6 months after intra-muscular injection of Al oxyhydroxide adjuvant in adult mice (Figure 2) [164]. Furthermore, neonatal injection of an Al oxyhydroxide-containing HBV vaccine provokes a Th2 immune reaction in the periphery, while raising IL-1β, IL-6, and tumor necrosis alpha (TNF-α) in the hippocampus and impairing hippocampal synaptic plasticity, whereas neonatal Bacille Calmette–Guérin (BCG) immunization provokes the opposite effect [193]. Al-containing hepatitis B injection during the first three weeks of life induces a proinflammatory profile of cytokine expression in the hippocampus [207], whereas in the periphery, it causes increased IL-4 levels and decreased proinflammatory cytokines [208].

Finally, a recent publication showed that immune regulatory molecules were significantly elevated in the brain after a peripheral immunological challenge induced after early injection of Al-containing vaccines or Al oxyhydroxide alone in rats [183]. Moreover, the impact of gender and genetic background on cytokine and chemokine reactions, along with astrogliosis and microgliosis in the brain, is highlighted in this study.

### 4.4. Autophagy Modulation by Al Particles

Besides its role in synaptic pruning and neurodevelopment, xeno/autophagy also has a central role in the cellular degradation mechanism of mineral particles incorporated by endocytosis [66,209]. In mouse and zebrafish studies, exposure to Al nanoparticles leads to alterations in the expression of autophagy-related proteins, associated with behavioral impairment, sometimes in a particle size-dependent manner [210,211]. Like other metals, nanomaterials, and metallic nanomaterials, ABAs exert some inhibitory effects on the autophagic process through lysosomal destabilization [205,212,213,214]. Biopersistence of Al could be related to its lysosome-destabilizing action, which could be caused by direct crystal-induced fracture of phagolysosomal membranes [123,205,214]. Once particles are transferred within double-membrane autophagosomes and then fused with reconstructed and re-acidified lysosomes, Al is exposed to lysosomal acid pH, being the only condition capable of dissolving Al particles [123].

MMF patients’ biopsies revealed that Al adjuvant particles inside macrophages are at least partially surrounded by a layer resembling an autophagosomal membrane [21,89,215]. The phagophore’s recruitment of the cytosolic light chain protein 3 (LC3) results in the formation of an autophagosome, a vesicular double membrane that engulfs ingested particles as well as mitochondria and peroxisomes [185,216].

Preliminary data on in vitro culture of MMF patients’ PBMCs exposed to Al oxyhydroxide or Al-adjuvanted vaccines revealed higher levels of a key protein able to negatively regulate autophagy [217].

### 4.5. Al, Autophagy, and BBB

Al and especially Al^3+^ ions can easily cross the BBB and/or damage its integrity, mainly by inducing oxidative stress [218,219,220]. Cerebral translocation of Al particles is significantly increased in mice with a chronically altered BBB [146]. Furthermore, it was shown in mice that nanoalumina particle exposure through the carotid artery could lead to (i) particle deposition in brain endothelial cells and accumulation in brain, (ii) increased autophagy-associated gene expression and autophagic activity in the brain, (iii) decreased tight junction protein expression, and (iv) increased BBB permeability, suggesting that autophagy is a key mechanism in nanoalumina-mediated neurovascular toxic effects within the CNS [221].

### 4.6. ABAs and ASD: What Are the Facts?

**Figure 3:**
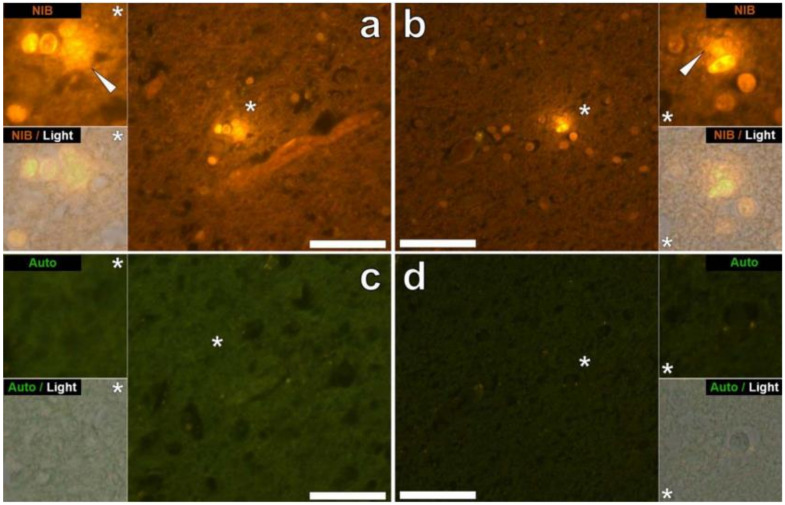
Intracellular aluminum in cells morphologically compatible with microglia within the parietal and temporal lobes of 29-year-old (A8) and 15-year-old (A4) male donors diagnosed with autism. Lumogallion-reactive extracellular aluminum (white arrows) producing an orange fluorescence emission was noted around likely microglial cells in the parietal (**a**) and temporal lobes (**b**) of donors A8 and A4, respectively. Non-stained adjacent (5 μm) serial sections produced a weak green autofluorescence emission of the identical area imaged in white (**c**) and gray matter (**d**) of the respective lobes. Upper and lower panels depict magnified inserts, marked by asterisks, of the fluorescence channel and brightfield overlay. Magnification ×400, scale bars: 50 μm. Copyright Elsevier (2018) [224].

ABA exposure during the first months of life presents two risks: direct particle toxicity to immune and nervous systems, and adverse effects caused by the activation of immune responses during these critical periods of development. In particular, the multiple hit model previously described in the present review seems to be adapted to the multiple consecutive immune activations due to the vaccination schedule in the first weeks/months of life [206]. Several studies have thus proposed that ABA exposure may be insidiously harmful for certain children over the short and long term, contributing to the tremendous increase in NDDs, especially ASD, at a young age [23,108,154,163,167,169,208,219,222,223,224,225] (for a review, see [226]). The main arguments in favor of this hypothesis are the following observations:

In human studies, on the one hand:

1. Tomljenovic and Shaw [227] described that the increased exposure to ABAs correlated with increased ASD incidence in the United States, and that the quantity of Al provided at 3–4 months of age was associated with ASD incidence in seven Western countries. In addition, postponing the first hepatitis B vaccination from the neonate stage (1st month of life) could reduce the incidence of ASD diagnosis in males by three times [228]. Because the infants in this study were born before the removal of mercury from vaccines, doubts persist about the particular impact of early mercury, Al, or the combination of mercury and Al exposure in ASD.

2. Mold and colleagues [224] used transversely graphite furnace atomic absorption spectroscopy to determine the Al concentration in post-mortem ASD brain tissue. Means from 2.30 to 8.74 μg Al^3+^/g were encountered, representing some of the highest values for Al in human brain tissue yet recorded. Moreover, this study also revealed Al intracellular localization in microglia-like cells and other inflammatory non-neuronal cells in the meninges, vasculature, gray, and white matter (Figure 3) [224]. Such observations can only be made post-mortem and do not allow the identification of the precise environmental origin of Al. However, they provide clues on shared cellular mechanisms - such as immune excitotoxicity - between neuronal Al content and ASD.

On the other hand, preclinical studies in rodent models clearly demonstrated that increased immune reactions during early life can induce autism-like behaviors [41,42,43]. These immune reactions can be caused by a toxic effect on the immune system during development or by an early intense (and/or repeated) stimulation of it. Mouse model studies during the early stages of postnatal development showed that Al adjuvants can adversely impact social behavior [163,167,169]. It was recently reported that intraperitoneal injection of hepatitis B vaccine during the first three weeks of age interfered with the developing mouse brain (impaired behavior, hippocampal long-term potentiation, decreased neurogenesis, microglial activation, and proinflammatory profile of cytokine expression in the hippocampus), probably mediated by IL-4 [207,208]. In addition, an adult sheep model also demonstrated that following repetitive shots of ABAs or ABA-containing vaccines, animals exhibited abnormal behaviors, such as increased aggressiveness and stereotypies, and decreased affiliative social interaction [168]. Furthermore, the reported increased levels of IL-6 in the blood and brains of rodents exposed to ABAs [183,193,194] are of particular interest due to the role of this interleukin as an important mediator of autism-like behaviors [60].

As a result of these pieces of evidence (epidemiological, clinical and preclinical data) pointing to a potential causal association between early ABA exposure and increased ASD risk [226], new hypotheses regarding neurological and immunological consequences of ABA-containing vaccines and novel clinical strategies (i.e., postponing of ABA-containing vaccines and replacement of ABAs with calcium phosphate are now being considered [225,229]. (Calcium phosphate was used in France until the mid-1980s mainly for the diphtheria-pertussis-tetanus vaccine group, without any mention of adverse reactions by physicians. Until the early-1970s it was also successfully used in pentavalent human vaccinations (smallpox, yellow fever, measles, BCG, and tetanus), also without any reported adverse reactions [230]. For a review on calcium phosphate, see [231]).

Meanwhile, vaccination during pregnancy is currently recommended worldwide (for instance, against pertussis), sometimes for each pregnancy [232,233,234]. Five pertussis vaccines are currently available, all with ABAs (Al oxyhydroxide, Al phosphate, or a mix of both) [234,235]. With regard to ABA neurodevelopmental toxicity discussed in this review, this prenatal exposure raises questions in addition to the previously discussed maternal immune activation model, related to (1) the very low number of studies focusing on prenatal exposure to ABAs (to our knowledge, so far only two studies on rats prenatally exposed to Al-containing HPV vaccine or to the adjuvant Al hydroxyphosphate sulfate have been performed until now, both by the vaccine industry [181,182]); moreover, (2) placental transfer to fetus tissues (including the brain) of Al compounds, in both humans and animals, was described [236,237,238,239], whereas there are no clinical data available addressing this question specifically for ABAs.

Considering that (i) autophagy is likely to be disrupted in ASD brains, (ii) this disruption could impair ABA clearance, (iii) ABAs are persistent pro-inflammatory particles in the early environment of babies, (iv) ABAs might significantly translocate to the brain with an immature BBB, altering the autophagy process and promoting neuroinflammation, and (v) that there is no proof to support that ABAs are completely secure to use in the children, we believe that further research should address the potential link between ABAs and NDDs [240,241], including:

1. Epidemiological studies comparing different vaccination schedules and ABA exposure in children;
2. Genetic studies of populations at risk, potentially targeting candidate genes in immune and autophagy pathways;
3. Pharmacokinetics and pharmacodynamics of Al adjuvants during both pre- and postnatal periods, in animal models;
4. Fundamental immunological data in order to better understand the mechanisms of immune action of ABAs, in addition to their possible capacity of inducing neuroinflammation and alterations of immune-neural interactions during early postnatal life, e.g., using autophagy-deficient mouse models.

## 5. Conclusions

The present review focused on ASD, yet the link between autophagy impairment, neurodevelopment, and early exposure to Al adjuvants could hold true for other neuropsychiatric disorders, including SZ and BD, now considered pieces of the same puzzle rather than separate entities (sharing clinical manifestations, vulnerability genes, and mechanisms) [242,243,244].

Our review presents the lack of fundamental scientific data demonstrating that Al adjuvants are safe and do not induce any long-term side effects. It also supports further investigation related to the effects of early Al adjuvant exposures occurring in combination with genetic susceptibility factors, including autophagy, immune and inflammation process genes. As accumulating evidence shows that modulating the levels of autophagy may increase the risk of NDDs, such studies will elucidate a new etiology for these complex disorders and contribute to develop potential new diagnostic and therapeutic tools.

## Abbreviations

ABAs	aluminum-based adjuvants
Al	aluminum
ASD	autism spectrum disorder
ASIA	autoimmune/inflammatory syndrome induced by adjuvants
BBB	blood–brain barrier
BD	bipolar disorder
CDC	U.S. Centers for Disease Control and Prevention
CNS	central nervous system
CNV	copy number variant
GxE	gene × environment
HBV	hepatitis b virus
IL	interleukin
ME/CFS	myalgic encephalomyelitis/chronic fatigue syndrome
MIA	maternal immune activation
MMF	macrophagic myofasciitis
MMR	measles, mumps, rubella
mTOR	mammalian target of rapamycin
NNDs	neurodevelopmental disorders
PBMCs	peripheral blood mononuclear cells
SZ	schizophrenia
WHO	World Health Organization
