# Correction: Angrand et al. Inflammation and Autophagy: A Convergent Point between Autism Spectrum Disorder (ASD)-Related Genetic and Environmental Factors: Focus on Aluminum Adjuvants. *Toxics* 2022, *10*, 518

**DOI:** 10.3390/toxics11020095

**Published:** 2023-01-19

**Authors:** Loïc Angrand, Jean-Daniel Masson, Alberto Rubio-Casillas, Marika Nosten-Bertrand, Guillemette Crépeaux

**Affiliations:** 1 Univ Paris Est Créteil, INSERM, IMRB, F-94010 Créteil, France; 2 Ecole Nationale Vétérinaire d’Alfort IMRB, F-94700 Maisons-Alfort, France; 3 INSERM UMR-S 1270, 75005 Paris, France; 4 Sorbonne Université, Campus Pierre et Marie Curie, 75005 Paris, France; 5 Institut du Fer à Moulin, 75005 Paris, France; 6 Biology Laboratory, Autlán Regional Preparatory School, University of Guadalajara, Autlán 48900, Jalisco, Mexico; 7 Autlán Regional Hospital, Health Secretariat, Autlán 48900, Jalisco, Mexico

## Text Correction

There was an error in the original publication [1]: “These salts are found in approximately 60% of humans (…)”.

A correction has been made to Section 4.1, second paragraph.

Corrected sentence is: “These salts are found in approximately 60% of human vaccines [106]”.

The authors state that the scientific conclusions are unaffected. This correction was approved by the Academic Editor. The original publication has also been updated.
